# Guidelines for Collection of Biological Samples for Clinical and Forensic Toxicological Analysis

**DOI:** 10.1080/20961790.2016.1271098

**Published:** 2017-01-16

**Authors:** Ricardo Jorge Dinis-Oliveira, Duarte Nuno Vieira, Teresa Magalhães

**Affiliations:** a Department of Legal Medicine and Forensic Sciences, Faculty of Medicine, University of Porto, Porto, Portugal; b Department of Sciences, IINFACTS – Institute of Research and Advanced Training in Health Sciences and Technologies, University Institute of Health Sciences (IUCS), CESPU, CRL, Gandra, Portugal; c UCIBIO-REQUIMTE, Laboratory of Toxicology, Department of Biological Sciences, Faculty of Pharmacy, University of Porto, Porto, Portugal; d Faculty of Medicine, University of Coimbra, Porto, Portugal

**Keywords:** Forensic science, forensic toxicology, samples collection, guidelines, kits and labelling, storage and preservation

## Abstract

This article aims to review general procedures for sampling of routinely collected as well as on alternative samples that may provide additional information regarding intoxication. These approaches may be applied whenever sample collection for clinical and forensic toxicology is required and should be considered as general guidelines that must be adapted to each specific case. It is expected that this article will help toxicologists and other forensic experts to accomplish their mission, since the toxicological result is first influenced by the quality and quantity of the sample available for analysis. These guidelines were approved by the European Council of Legal Medicine.

## Introduction

There are several specific challenges to select and collect samples for *antemortem* and *postmortem* toxicological analysis [1]. Indeed, the relevance of any finding is primarily determined by the type and quality of the sample(s) submitted to the laboratory. Thus, numerous pre-analytical aspects should be considered during collection in order to have samples with sufficient quality and quantity. In several cases, the evidence found at the scene may represent the best guide for toxicological analysis and therefore cups, bottles, pipes, syringes/needles, cotton, lemon, spoons, silver paper and suspicious household products should be collected.

*In vivo*, blood, plasma or serum, urine, hair, nails, exhaled air, oral fluid and gastric content are the most important samples to be collected and should be obtained at the hospital admission, before the implementation of the therapeutic measures [2,3].

*Postmortem* samples may be numerous and present specific difficulties compared to *in vivo* samples, namely those resulting from putrefactive alterations. Arterial or venous femoral and cardiac blood, urine, vitreous humour, gastric content and organs (namely the liver and lungs and always after evisceration) are the most important samples to be collected [2,3]. Several other alternative samples (e.g. blood clots, blood from thoracic or abdominal cavities, cerebrospinal fluid, brain, spleen, bile, bone, synovial fluid, bone marrow, maggots, skeletal muscle) can be occasionally collected in particular circumstances, which are detailed below in specific sections [2,3].

Relevant quantitative analysis is usually only possible using plasma, serum or blood (i.e. obtained from specific comportments). For these samples, numerous methods for analysis and *antemortem* and *post-mortem* casuistic data are available to stablish correlation of obtained concentrations and the toxic effects. However, in several cases plasma, serum or blood are simply not available or they are present with low quality. Moreover, the site of blood collection for *post-mortem* toxicology may influence xenobiotic concentrations. Therefore, toxicologists are constantly looking for alternative samples that accurately represent *post-mortem* blood drug concentrations or that may be valuable in clarifying blood results [2,3].

Nowadays, pre-analytic best practices for sample collection in Forensic and Clinical Toxicology are scarce, not updated or are not presented objectively to avoid errors. Moreover, most of them cover only few number of samples. This work presents guidelines for sample collection for toxicological analysis that were approved by the European Council of Legal Medicine (http://www.eclm.info/html/).

## Methods

Literature in Forensic, Clinical and Analytical Toxicology and Pathology was extensively reviewed since 2000, aiming to understand procedures for sample collection followed/advised by authors publishing in this area. Websites of Associations of Forensic, Clinical and Analytical Toxicology were also reviewed, such as The International Association of Forensic Toxicologists [4], German Society of Toxicological and Forensic Chemistry [5] and Society of Forensic Toxicologists /American Academy of Forensic Sciences [6]. General relevant literature can be found in Refs. [1–13] and additional specific citations are provided in the sections below.

## Procedures for collection

It is obvious that samples should be collected without delay after death. If autopsy cannot be performed within few hours after death, mortuary refrigeration is usually the unique procedure to minimize alterations in the concentration due to putrefactive changes. Indeed, even anticipating autopsy permission, most jurisdictions restrict any cadaver interventions. If possible, basic procedures such blind femoral blood collection through the body surface or urine by catheter or even suprapubic puncture may be implemented [11]. In the following sections, sampling procedures for bioanalysis are provided tissue-by-tissue, together with notes and comments on the value and interpretation of results. Specifically, the anatomic place of collection, tubes to be used, storage, advantages and limitations and amounts that should be collected are discussed for *in vivo* and *post-mortem* samples.

### In vivo samples

1. Exhaled air:
  - Should be collected for volatile compounds analysis such as ethanol and in carbon monoxide poisoning. Several other xenobiotics (e.g. amphetamines, benzodiazepines, cocaine, opioids, etc.) are being analysed, possibly increasing the future application of this sample [14–16];
  - non-invasive;
  - large volume available;
  - useful for both qualitative and quantitative analysis.
2. Oral fluid [1]:
  - Collect 1–2 mL for an appropriate plastic container (with preservative);
  - several collection devices are available in the market;
  - it should be considered that the sample can be diluted due to buffer, preservative or other reagents present in the collection devices;
  - useful for most xenobiotics, namely drugs of abuse (e.g. roadside testing);
  - good correlation exists with the blood concentrations of free and non-ionized xenobiotic fractions and therefore with toxic effects;
  - due to possible external contamination or depot (e.g. drugs smoked or administered sublingual and *per os*) may not accurately reflect blood xenobiotic concentrations. To reduce this possibility, the donor should be observed for 10–15 minutes before collection without smoking, drinking or eating;
  - the detection window is short as blood;
  - rinsing the mouth is not required for collection and does not lower concentrations;
  - non-invasive (i.e. can be performed by the donors themselves) in opposition to venipuncture and can be obtained shortly after incident time;
  - the witnessed collection reduce any attempt of adulteration;
  - as any other biological fluid, care should be paid during handling due to the risk of infection;
  - less fluid than urine due to the content in mucopolysaccharides and mucoproteins;
  - may not be available in cases of xerostomia;
  - with due precaution, can be useful for quantitative analysis.
3. Breast milk:
  - Collect 30 mL for an appropriate plastic container (without preservative);
  - usually available in large amounts;
  - collection implicates invasion of privacy;
  - useful to estimate body burden of contaminants in adipose tissue;
  - useful to determine whether an infant was exposed to a drug through that route;
  - contains high concentrations of lipids and proteins, and the pH is slightly lower than plasma (6.35–7.35). However, intra (e.g. during the last fed portions, the lipid content increases) and interday variations in composition are observed during lactation, which may influence xenobiotic transfer and therefore obtained concentrations;
  - important excretion route for lipophilic xenobiotics in the lactating women, namely those with a molecular weight lower than 200 Da, low plasma protein binding and with basic pKa (undergo ion-trapping effect) [17].
4. Sweat:
  - Should be collected at least during one week (without preservative);
  - useful for workplace drug testing;
  - non-invasive;
  - collection is not easy and requires time to obtain an adequate volume for analysis;
  - only useful for qualitative analysis.
5. Amniotic fluid:
  - Collect 10 mL for plastic container with screw cap;
  - useful to evaluate intrauterine xenobiotic exposure;
  - minimal laboratorial sample preparation is required;
  - invasive collection that requires local anaesthetic, ultrasound scan and highly trained physician;
  - only useful for qualitative analysis.
6. Meconium [18]:
  - Collect all available (2 g minimum) for a plastic container (without preservative);
  - always collected in suspected uterine xenobiotic exposure;
  - higher xenobiotic's concentrations are expected than in urine due to accumulation during gestation;
  - easily collected from newborn compare to urine and hair is not always available;
  - large window to document prenatal exposure during the last two trimesters of pregnancy;
  - collection should be performed within 72 h;
  - only useful for qualitative analysis.

### Post-mortem samples

1. Cardiac blood:
  - Collect 30 mL for a plastic container with screw cap. Preservative is not mandatory;
  - always collected;
  - the sampling site should be mentioned in the label;
  - collection from right chamber is preferable;
  - useful for qualitative/screening analysis since large volumes are available;
  - concentrations of xenobiotics may increase due to *post-mortem* redistribution, autolysis of cardiomyocytes or trauma;
  - although quantitative analysis is possible, this should be discouraged or decided case by case.
2. Blood clots from subdural, subarachnoid and/or epidural spaces:
  - Collect 30 mL for a plastic container with screw cap (without preservative);
  - should be collected in traumatic cases and can be particularly useful in burned cases;
  - are potential “time capsules” since are generally less perfused spaces, and therefore may reflect xenobiotic concentrations closer to the time of injury (i.e. when the hematoma was formed);
  - the importance increases if victim survives several hours after the trauma and if injury time is accurately known;
  - only useful for qualitative analysis.
3. Blood from thoracic or abdominal cavities:
  - Collect 30 mL for a plastic container with screw cap (without preservative);
  - should be collected in traumatic cases, especially if no other blood or uncontaminated blood clots cannot be obtained;
  - strong possibility of contamination exists specially with micro-organisms, urine and gastric and intestinal contents;
  - only useful for qualitative analysis.
4. Pleural effusions [19]:
  - Collect 30 mL for a plastic container with screw cap (without preservative);
  - particular useful in cases exhibiting advanced putrefaction;
  - only useful for qualitative analysis.
5. Brain:
  - Collect 30 g for a plastic container with screw cap (without preservative);
  - relevant for drugs that act on the central nervous system;
  - collected for lipophilic (e.g. drugs of abuse, organochlorated insecticides, etc.) and volatile xenobiotic analysis;
  - concentrations of xenobiotics are not affected by *post-mortem* redistribution from the stomach or by the putrefaction of other organs (e.g. intestine, liver, lungs);
  - concentrations may vary significantly from one region to another, but current data fail to suggest that one area should be collected over another;
  - the high lipid content may cause analytical problems;
  - only useful for qualitative analysis.
6. Vitreous humour:
  - Collect all available (normally 2–5 mL in adults and 1 mL in newborns) for a 10 mL plastic container with screw cap (add preservative);
  - useful for the analysis of several psychoactive substances (e.g. ethanol) and biochemistry analysis;
  - it is easy to collect and remains clear and sterile for up to three days or so after death. It becomes cloudy and brown with decomposition;
  - it should be obtained by ophthalmocentesis from each eye. The needle should be inserted through the outer corner, until its tip is placed centrally in the globe and gentle aspiration should be performed to avoid retinal detachment;
  - due to limited available volume, the fluid from both eyes can be combined;
  - cosmetic integrity of eyes can be restored by injecting an appropriate amount of saline solution;
  - good correlation exists with the blood free xenobiotic concentration;
  - xenobiotics with high binding percentages to serum proteins will present much lower concentrations than blood;
  - concentrations lag behind blood levels approximately 1–2 h;
  - useful in the interpretation of blood results or when blood is not available (e.g. trauma);
  - if positive, it is useful to prove *antemortem* ethanol ingestion and therefore discarding *post-mortem* ethanol production by fermentation;
  - because the eye is distant from the major thoracic and abdominal organs and it is a closed space, it is less influenced by contamination, putrefaction and *post-mortem* redistribution;
  - may not become contaminated by embalming process. In these cases, it is important that a sample of embalming fluid should be submitted for analysis as control;
  - lacks esterases that hydrolyse and therefore reduce the blood concentrations of certain xenobiotics (e.g. cocaine, heroin, 6-monoacetylmorphine);
  - quantitative analysis is rarely possible (except ethanol) and should be considered with due care;
  - if positive for ethanol, it can be assumed that represents an *antemortem* consumption;
  - if negative for ethanol, but blood samples are positive, ethanol production in blood should be suspected due to *post-mortem* putrefaction;
  - it has a high water and low lipid content in comparison to blood. Therefore, concentrations of highly lipophilic xenobiotics in vitreous humour will be lower;
  - High *post-mortem* glucose concentrations suggest *peri-mortem* hyperglycaemia and if conjugated with elevated acetone levels, suggests diabetic ketoacidosis. Due to the rapid *post-mortem* decrease of “normal” glucose concentrations, low or even “zero” levels may erroneously suggest hypoglycaemia.
7. Spleen:
  - Collect 30 g for a plastic container with screw cap (without preservative);
  - useful when blood is not available such in fire-related deaths and for xenobiotic that accumulate in erythrocytes (e.g. carbon monoxide and cyanide);
  - only useful for qualitative analysis.
8. Lung:
  - Collect 30 g for a plastic container with screw cap (without preservative);
  - collected for volatile xenobiotics (e.g. toluene, nitric oxide, butane): the main bronchus is tied off tightly with a ligature, the hilum is then divided and the lung put into a glass container, which is sealed with polytetrafluoroethylene (Teflon®) or aluminium foil-lined lids and immediately sent for analysis. Plastic containers are not suitable, since greater diffusion of volatiles is expected;
  - for other xenobiotics (e.g. paraquat) the apex of the right lung is preferable. The basal lobes, namely of the right lung, are more prone to *post-mortem* redistribution from gastric contents due to stomach proximity. Moreover, due to high blood perfusion, it is likely to have an important contribution for *post-mortem* redistribution;
  - only useful for qualitative analysis.
9. Liver:
  - Collect 30 g for a plastic container with screw cap (without preservative);
  - always collected;
  - identify source – deep right lobe is preferred to avoid contamination with diffusion of xenobiotics from gastric contents into the left lobe;
  - gall bladder should not be collected together with liver;
  - useful for almost all xenobiotics since it is the major metabolic organ and an important depot (e.g. tricyclic antidepressant), making at least the qualitative analysis for certain xenobiotics easier than in blood in some cases;
  - although very difficult to become routine, if quantitative analysis is required, this is the most promising solid tissue since more data exist for liver xenobiotic concentrations than for any other organ;
  - quantitative relationships between liver and blood concentrations for most xenobiotics are not available;
  - since concentrations of xenobiotics do not change markedly *post-mortem* (at least in the early period), ratios for peripheral blood have been proposed either as markers of *post-mortem* redistribution and to stablish correlations:
  - ratios lower than 5 suggest low or even none propensity for *post-mortem* redistribution;
  - ratios exceeding 20–30 suggest significant *post-mortem* redistribution;
  - the high lipid and protein content may cause interferences in toxicological analysis.
10. Bile:
  - Collect all available for a 10 mL plastic container with screw cap (without preservative);
  - particularly useful when urine is absent and in cases of long survival after last administration;
  - important for xenobiotics that exhibit enterohepatic circulation (i.e. those extensively conjugated with glucuronic acid, glutathione or sulphate) and for chronic exposures (e.g. opioids, cannabinoids, benzodiazepines, etc.);
  - it is a relatively “dirty” fluid, containing high concentrations of bile salts and other substances that may interfere with toxicological analysis;
  - the gallbladder is tied to reduce contamination and bile is collected by aspiration or directly from the common bile duct if cholecystectomy was performed. Always collected prior to liver;
  - concentrations may be altered by *post-mortem* diffusion from the liver and the stomach;
  - only useful for qualitative analysis.
11. Kidney:
  - Collect 30 g for a plastic container with screw cap (without preservative);
  - collected for heavy metals (tend to concentrate in the kidneys) or ethylene glycol analysis;
  - capsule should be removed;
  - could be important in the absence of urine;
  - only useful for qualitative analysis.
12. Heart:
  - Collect 30 g for a plastic container with screw cap (without preservative);
  - left ventricle should be considered;
  - not very useful; acquires some importance in the interpretation of blood concentrations in digitalis intoxications;
  - only useful for qualitative analysis.
13. Bone:
  - Collect 30 g for a plastic container with screw cap (without preservative);
  - collected from skeletonized remains;
  - should be cut into small pieces (e.g. femur rings) or crushed;
  - there are no data to suggest that one anatomic region is better than another. Larger bones (e.g. femur) are certainly easier to work/extract analytes with than smaller bones;
  - both intra and interbone differences in xenobiotic distribution are possible [20];
  - only useful for qualitative analysis.
14. Synovial fluid:
  - Collect all available from each uninjured joint cavity for a 5 mL plastic container with screw cap (add preservative) [21];
  - it is usually obtained by lateral puncture of the bursa sac under the patella. Approximately 1–2 mL can be collected, on the average, by each puncture and can be combined;
  - care should be taken to avoid rupture of the bursa sac;
  - does not introduce cosmetic alterations;
  - similarly to cerebrospinal fluid and humour vitreous, it is in an isolated compartment protected by the bursa sac with firm tissue structures and therefore much less influenced by putrefactive changes or *post-mortem* redistribution of xenobiotics;
  - rarely used. Acquires some relevance when putrefactive changes are relevant and humour vitreous is unavailable (e.g. due to trauma or heat exposure);
  - it is more viscous than vitreous humour rendering analysis more difficult;
  - only useful for qualitative analysis but relevant quantitative results were already obtained for ethanol and do not contain alcohol dehydrogenase [22,23].
15. Bone marrow:
  - Collect all available for a 10 mL plastic container with screw cap (without preservative);
  - useful in advanced stages of putrefaction since it is protected by bones;
  - high degree of vascularity and lipid content (may accumulate lipophilic xenobiotics);
  - different xenobiotic concentrations are registered in the marrow obtained from the same or different bones [24];
  - in xenobiotic distributions are possible [20];
  - the label should detail the sampling site;
  - typically, the ribs are cut approximately 5 cm far from its distal end (i.e. near the medioclavicular line) where it is ossified. By compressing/squeezing the remaining rib ends using pliers, the dark red bone marrow can be aspirated;
  - although less common, it can be obtained by trocar aspiration from the vertebral bodies (*antemortem*) or from femur after section of the cortical bone (*post-mortem*);
  - correlation with blood results are no easily interpreted;
  - only useful for qualitative analysis.
16. Fly larvae (maggots) – entomotoxicology:
  - Collect 10 larvae randomly collected of an organ for a plastic container with screw cap (without preservative);
  - useful in advanced stages of putrefaction, when conventional samples are not available;
  - the xenobiotic concentrations depend on the tissue that served as food for the larvae, as well as varies interspecies and intraspecies during their stage of development;
  - the organ of collection should be identified;
  - significant loss in xenobiotic concentration occurs within one day after larvae has been removed from tissue; therefore, feeding larvae is the most desirable insect stage for collection;
  - insects remain (e.g. puparia or exuviae) may persist for a long time, even when classical samples are no longer available;
  - only useful for qualitative analysis.
17. Adipose tissue:
  - Collect 30 g for a plastic container with screw cap (without preservative);
  - the anatomical site for collection leads to unpredictable results, but abdominal subcutis has been more analysed;
  - it is rarely useful. Nevertheless, since it acts as a reservoir, it can be collected for lipophilic xenobiotics especially if preferable samples are absent;
  - is not be frequently analysed due to the difficult analysis and variability of xenobiotic concentration from one site to another;
  - xenobiotics’ detection reflects *antemortem* accumulation and not the result of *post-mortem* redistribution;
  - only useful for qualitative analysis.
18. Skin:
  - Collect the skin (radius of approximately 2–4 cm around needle puncture or chemical burn) for a plastic container with screw cap (without preservative);
  - a distant, preferably contralateral area, should be collected as a control sample for different container. However, caution should be paid since alternate sides may be used as occurs in drug dependence.
19. Skeletal muscle:
  - Collect 30 g for a plastic container with screw cap (no preservative);
  - useful in advanced decomposition cases since it is more resistant to autolysis;
  - it is available in large amounts;
  - normally collected from right or left lumbar portion of the iliopsoas muscle;
  - different aliquots obtained from the same muscle collected from the same leg may lead to different concentrations;
  - results interpretation is difficult;
  - only useful for qualitative analysis.

### Antemortem or post-mortem samples

1. Peripheral blood:
  - Collect 10 mL for a plastic container with screw cap. A preservative fluoride salt (such as sodium or potassium; 1%–5%) is mandatory;
  - an aliquot without preservative should be saved, namely in cases of suspicion of fluoride poisoning;
  - always collected for complete toxicology analysis;
  - reduced volume and more difficult to collect than cardiac blood;
  - *post-mortem* – venous and/or arterial femoral blood should be collected since it is relatively isolated from the internal organs of the chest and abdomen and therefore less influenced by the *post-mortem* redistribution phenomenon. Vessel should be tied/clamped proximally near the inguinal ligament before collection to prevent siphoning blood down from the larger central vessels. The leg may be slightly elevated to obtain more blood and should not be massaged or shaken to increase flow. Alternatively, venous subclavian or jugular blood can be collected. In any case, a “blind-stick” will increase the probability of false results;
  - *antemortem* – although it is homogeneous throughout all anatomic places, blood is typically collected from cephalic vein. Cord blood is obtained from the umbilical cord at parturition;
  - do not use antiseptic swabs containing alcohols or iodine to disinfect the skin prior to venipuncture, since blood contamination can occur, especially when performing ethanol analysis;
  - should never be mixed with cardiac blood;
  - for analysis volatile xenobiotics, a gas syringe should be used;
  - it is the most useful sample for quantitative analysis, but usually analysis gives total fraction (i.e. free and bound xenobiotic to proteins). Quantification of free fraction would be useful, but is rarely possible.
2. Urine:
  - Collect 30 mL or all available for a plastic container with screw cap (without preservative);
  - always collected for screening (higher concentrations of the parent xenobiotics and metabolites are usually found in urine comparatively to other sample), acquiring great value if obtained up to 96 h after intoxication;
  - *post-mortem* collection can be performed by inserting the needle directly above the pubic symphysis or by urethral catheterization (in cases of external examination only) or directly from the bladder (if internal examination is also performed);
  - if bladder contains only small amounts of urine, it may be necessary to open bladder to collect any residual quantity. If urine is not available, the bladder could be washed with a saline solution in order to obtain any sample and it must be assured that vitreous humour is provided [10];
  - results could be negative if death occurred closely the time of intoxication;
  - ensure that the sample actually comes from the patient being examined to avoid adulteration;
  - no correlation exists with blood levels; higher concentrations are usually present;
  - No correlation exists with toxic effects at the time of collection; bladder is primarily a reservoir and unlike blood, is not in equilibrium with tissues;
  - for ethanol analysis in a type I and II diabetic victims and if urine is contaminated with *Candida albicans* (e.g. immunosuppressed victims or with gynaecological and urinary infections), preservative may be useful;
  - quantitative analysis is rarely possible.
3. Head hair [7,25]:
  - Collect 150–200 hairs (often described as having a pencil thickness) from the posterior vertex region of the scalp. This anatomic place presents the least growth variation in comparison to other regions of the scalp and other body hair types. Collection from multiple sites within the vertex region is acceptable to avoid a visible “bald patch” that may cause some discomfort;
  - if it is not available or excessively bleached or permed, axillar, pubic, arms or bear hair is an alternative;
  - plucked in *post-mortem* (prior organ dissection) or cut with scissor just near the root *in vivo* cases;
  - samples should be firmly tight together and tie with ligature to not loose orientation (the root should be identified);
  - the sample is placed on a piece of aluminium foil, which is folded, with the cut root ends projecting about 15 mm beyond the end of the foil;
  - it is important to avoid folding in the middle to not kink the hair making it difficult to handle;
  - should be considered to establish a historical pattern of xenobiotic exposure (e.g. drug facilitated sexual aggression, habit changes, heavy metals chronic exposure, etc.);
  - it is not a suitable sample to document recent xenobiotic exposure;
  - it is not easily adulterated and new and identical sample can be obtained from the subject for counter-proof (e.g. sample switching, break in the chain of custody, etc.);
  - store at room temperature in a sealed envelope;
  - large detection window (i.e. weeks to months, depending on the hair length);
  - resistant to decomposition, being particularly useful in exhumed cadavers;
  - external contamination may cause false positive results if decontamination is not performed during analysis;
  - only useful for qualitative analysis.
4. Gastric content (including vomit and gastric aspirate):
  - Collect 30 mL aliquot of the total homogenized for a plastic container with screw cap (without preservative);
  - if it is absent, collect 30 g of the stomach wall (without preservative);
  - usually collected for complete toxicology testing;
  - the distal oesophagus and the pylorus should be clamped before the stomach is removed. Then, the stomach wall is cut and all the content is collected;
  - only all quantity is important and not the concentration;
  - register all total volume in the container's label;
  - macroscopic findings (e.g. tablets and capsules) should be rapidly separated, dried and stored in a different container;
  - characteristic odours should be registered, but if cyanides or phosphides gases are suspected to have been ingested caution should be paid to avoid inhalation;
  - useful to guide blood analysis;
  - presents limited application if intoxication occurred by parenteral route;
  - the presence of a xenobiotic in the gastric content does not prove oral administration, especially if the concentration is low. Indeed, xenobiotics distributed by the extracellular fluid will be presented in the fluid that ultimately forms the gastric secretions. Moreover, basic xenobiotics will be concentrated in the stomach due to the ion-trapping effect.
5. Cerebrospinal fluid:
  - Collect all available for a 10 mL plastic container with screw cap (without preservative):
  - presents similar composition to plasma except that high molecular weight proteins are absent;
  - *Antemortem* or *post mortem* it is obtained by percutaneous lumbar (by passing a needle into the theca between the lumbar spines) or cisternal (through the atlanto-occipital membrane) puncture, preferably before the internal examination. The lack of any intrathecal pressure may turn this collection very difficult or even impossible. Alternatively, it can be obtained from the posterior fossa after the brain has been removed; this procedure may lead to alterations of the xenobiotic concentrations due to blood contamination. Clear cerebrospinal fluid may, sometimes be obtained from the lateral ventricles, either by needle puncture reflecting the dura and parting the cerebral hemispheres or cutting down through the cortex [10,26];
  - xenobiotic concentrations are generally higher in blood;
  - correlation with blood xenobiotic concentrations;
  - only useful for qualitative analysis.
6. Finger and toe nails [27]:
  - *Antemortem* nail clippings should be obtained from all fingers and toes (and combined) using Teflon®-coated stainless steel scissors to reduce contaminations;
  - *post-mortem* all nails should be lifted from the fingers and toes;
  - Length growth rates were reported to be 3–5 mm and 1.1 mm *per* month for the fingers and toes, respectively [28];
  - easy to store (room temperature);
  - external contamination may cause false positive results if decontamination is not performed during analysis;
  - useful in decomposed bodies when conventional samples are not available;
  - retrospective/past window detection of xenobiotics, even potentially longer than hair; the hallux nail may document as long as 12 months of exposure;
  - formation begins during the second trimester of gestation. Therefore, it is useful to prove intrauterine xenobiotic exposure;
  - it is a discharged material and xenobiotic incorporation is not influenced by melanin content;
  - only useful for qualitative analysis.

## Kits: containers, labelling and toxicological request form

There is considerable variation in the types of kits used by forensic institutions. Regardless the format, it is key to the successful collection and consequent toxicological result to have necessary sample containers, to ensure that they are adequately labelled and that chain of custody is respected. The following topics could be highlighted [1,2,11,12,26]:

- Biological fluids can be collected using either wide-bore pipettes or disposable hypodermic syringes with appropriate needle gauges and lengths [2];
- contamination may be introduced if metal scalpels or needles are used for collection and metal analysis is subsequently performed;
- containers should be new and preferably rinsed with distilled water and sterilized before use, unless the manufacturer's states it unnecessary;
- separate containers should be used to accommodate different samples and plastic (especially polypropylene) with screw caps is useful in the majority of cases since it does not break, especially during frozen. If volatile xenobiotics (e.g. solvent abuse or intoxication with anaesthetic gases) are to be analysed, samples should be promptly collected and glass containers sealed with polytetrafluoroethylene (Teflon®) or aluminium foil-lined lids are preferable to avoid greater losses by diffusion registered through plastic containers [29];
- containers should be filled (but not overfilled) to minimize headspace and therefore losses due to evaporation (e.g. volatiles such as ethanol);
- containers should be open at the time of analysis and only when cold at 4 °C;
- a self-adhesive tamper-resistant stickers should be placed over container lids to assure that samples were not adulterated;
- as a minimum, the label should include the following information: institutional case number identifier or request number; name of the victim or other identifier; sample type (e.g. blood, liver, kidney, etc.) and anatomic place of blood collection when applicable (e.g. cardiac *versus* femoral blood); signature of the examiner; date and time of collection [2];
- toxicological request forms should be filled as complete as possible, placed with samples inside a sealed plastic opaque bags and submitted to the laboratory for analysis;
- a chain of custody report should be completed and signed to evidence sample integrity.

## Sample preservation and storage

Putrefactive *post-mortem* alterations that occur in body are influenced by several variables and can dramatically influence the obtained concentrations for several analytes (e.g. blood ethanol concentrations may increase or decrease). Although these consequences are almost uncontrollable, further alterations in xenobiotic concentrations due to incorrect *in vitro* preservation and storage are less tolerated. Therefore, the preservation of samples and physical conditions (e.g. temperature) during storage should not be disregarded, since alterations in the concentrations of various analytes (e.g. blood ethanol concentrations) may occur, even *in vitro* [30]. In the following topics it is highlighted general procedures for sample preservation and storage [1,2,11,12,26]:

- Samples should be stored in tightly sealed containers at 4 °C (short-term) or at −20 °C or preferably at −80 °C (long-term);
- exceptions to this include hair and nail, which are stable at room temperature;
- hair samples should be stored at room temperature;
- if plasma or serum is needed for analysis, these are separated before blood frozen;
- sodium (or potassium) fluoride preservation of blood with a final concentration of 1%–5% by weight is mandatory for peripheral blood and facultative for other blood samples (e.g. blood clots and blood from thoracic or abdominal cavities). Clear evidence of the importance of preservative exists for several analytes such as opioids, ethanol, cocaine, cyanide, γ-hydroxybutiric acid, etc.

## Interpretation aspects

At the end of the technical note, some points must be remembered [1–3,7–9]:

- Forensic experts must be aware that it is mandatory the collaboration of a forensic toxicologist in the interpretation of toxicological results;
- although there is no a universally accepted standard education, the forensic toxicologist should be endowed with adequate training in analytical chemistry, but specially depth knowledge of the toxicokinetic and toxicodynamic of xenobiotics, and be attentive for the variables that can influence them;
- it would be advisable that the collection procedure of pathologists be audited by toxicologists to avoid errors;
- absolute rules for the interpretation of toxicological results are absent since each case is unique!
- it is almost impossible to know the true concentration of a xenobiotic in a sample;
- attempts to interpret toxicology findings exclusively based on the therapeutic, toxic, and fatal ranges are irresponsible, especially in *post-mortem* toxicology. These intervals frequently overlap;
- the case should be interpreted taking into account *antemortem* or *post-mortem* toxicological results, circumstances of the death and scene, medical history, autopsy findings, influence of *post-mortem* redistribution, tolerance and application of common sense, etc.
